# Understanding implementation and usefulness of electronic clinical decision support (eCDS) for melanoma in English primary care: a qualitative investigation

**DOI:** 10.3399/bjgpopen18X101635

**Published:** 2019-03-20

**Authors:** Merel M Pannebakker, Katie Mills, Margaret Johnson, Jon D Emery, Fiona M Walter

**Affiliations:** 1 Research Associate, The Primary Care Unit, Department of Public Health and Primary Care, University of Cambridge, Cambridge, UK; 2 Research Associate, The Primary Care Unit, Department of Public Health and Primary Care, University of Cambridge, Cambridge, UK; 3 Patient Representative, c/o The Primary Care Unit, Department of Public Health and Primary Care, University of Cambridge, Cambridge, UK; 4 Herman Professor of Primary Care Cancer Research, Department of General Practice and the Centre for Cancer Research, Faculty of Medicine, Dentistry and Health Science, University of Melbourne, Victorian Comprehensive Cancer Centre, Melbourne, Australia; 5 Visiting Senior Researcher, The Primary Care Unit, Department of Public Health and Primary Care, University of Cambridge, Cambridge, UK; 6 Reader in Primary Care Cancer Research, The Primary Care Unit, Department of Public Health and Primary Care, University of Cambridge, Cambridge, UK; 7 Honorary Associate Professor, Department of General Practice and the Centre for Cancer Research, Faculty of Medicine, Dentistry and Health Science, University of Melbourne, Victorian Comprehensive Cancer Centre, Melbourne, Australia

**Keywords:** melanoma, skin cancer, qualitative, electronic clinical decision support (eCDS), patients, general practice

## Abstract

**Background:**

Timely diagnosis of the serious skin cancer melanoma can improve patient outcomes. Clinical guidelines suggest that GPs use checklists, such as the 7-point checklist (7PCL), to assess pigmented lesions. In 2016, the 7PCL was disseminated by EMIS as an electronic clinical decision support (eCDS) tool.

**Aim:**

To understand GP and patient perspectives on the implementation and usefulness of the eCDS.

**Design & setting:**

Semi-structured interviews with GPs and patients were undertaken. The interviews took place in four general practices in the south east of England following consultations using the eCDS for suspicious pigmented lesions.

**Method:**

Data were collected from semi-structured face-to-face interviews with GPs and from telephone interviews with patients. They were recorded and transcribed verbatim. The Consolidated Framework for Implementation Research (CFIR) underpinned the analysis using thematic approaches.

**Results:**

A total of 14 interviews with GPs and 14 interviews with patients were undertaken. Most GPs reported that, as the eCDS was embedded in the medical record, it was useful, easy to use, time-efficient, and could facilitate patient–GP communication. They were less clear that it could meet policy or patient needs to improve early diagnosis, and some felt that it could lead to unnecessary referrals. Few felt that it had been sufficiently implemented at practice level. More felt confident with their own management of moles, and that the eCDS could be most useful for borderline decision-making. No patients were aware that the eCDS had been used during their consultation.

**Conclusion:**

Successful implementation of a new tool, such as eCDS for melanoma, requires GPs to perceive its value and understand how it can best be integrated into clinical practice. Disseminating a tool without such explanations is unlikely to promote its adoption into routine practice.

## How this fits in

Clinical decision support tools have the potential to help identify people at risk of cancer for early referral and investigation, but there is little research evidence to guide their integration into clinical software. This qualitative interview study with GPs and patients found that, compared with a paper version, GPs viewed an embedded eCDS tool for suspicious pigmented lesions as easier to use, time-efficient, and having the potential improve communication in the consultation. Some felt that it could be more useful for borderline lesions but could lead to unnecessary referrals. These findings can support implementation of eCDS tools for melanoma and other cancers into routine clinical practice.

## Introduction

Melanoma is the leading cause of skin cancer deaths in the UK, causing 2285 deaths in 2016.^1^ It is associated with significant morbidity, and the thickness of the lesion at diagnosis is the most important prognostic factor; for example, stage 1 disease has 5-year survival rates of >95%, compared with 8% for males and 25% for females with stage 4 disease.^2^ Emerging evidence suggests that opportunities to diagnose cancer earlier in primary care may be missed owing to failure to consider the diagnosis, investigate it, or refer appropriately.^3,4^ Therefore, improving approaches to clinical assessment and management of patients with potential skin cancer could improve their diagnostic experiences and outcomes.^5^ In the NHS and similar healthcare systems, primary care is the first point of contact for most patients worried about a skin change. Diagnosing relatively rare conditions such as melanoma in primary care is challenging. This is because similar symptoms and signs are commonly presented and usually caused by benign conditions such as seborrheic keratoses and benign naevi.

Clinical decision support (CDS) tools have the potential to help identify patients at risk of cancer for early referral and investigation.^6–8^ However, while the aim of giving GPs tools to help them identify people with symptoms suspicious of cancer sooner is recommended by the National Institute for Health and Care Excellence (NICE),^9^ there is currently no clinical trial evidence to support their integration into clinical software, despite widespread dissemination in England.^10,11^ A common problem in the NHS is the introduction of change without sufficient piloting and attention to implementation, and there is little evidence of how cancer tools are used, their safety, any harms that may occur for patients and their GPs, and whether they have any positive impact on outcomes for patients.^12^ Feasibility and qualitative studies examining non-electronic^6,13^ and electronic^14^ forms of cancer CDS tools, and set in English primary care, demonstrated GP concerns about increased patient anxiety and inappropriate referrals, despite an increase in cancer diagnostic activity, urgent referrals, and cancer diagnoses.

**Figure 1.**
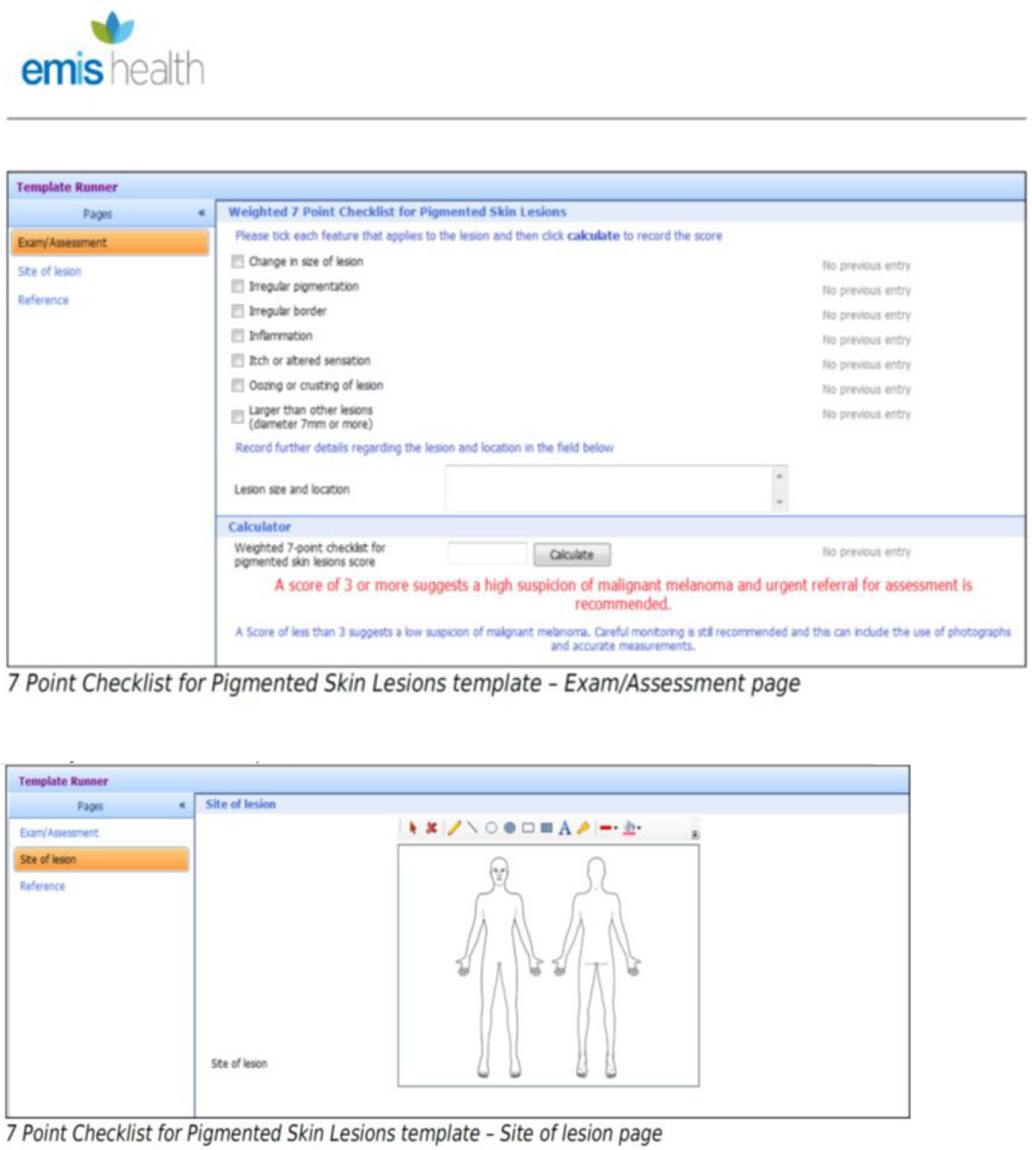
Screenshots of EMIS software to show electronic clinical decision support for melanoma. eCDS = electronic clinical decision support.

Computerised symptom checklists may be easier to implement than more sophisticated cancer decision support tools such as QCancer. For suspicious pigmented lesions, the NICE guidelines for suspected cancer recommend GPs take a history and undertake naked-eye examination guided by the weighted 7PCL.^8^ This checklist consists of three major features (scoring 2 points each): change in size; irregular shape; and irregular colour. It also consists of four minor features (scoring 1 point each): diameter greater than 7mm; inflammation; oozing; and change in sensation. The paper version of the 7PCL has been validated as a diagnostic aid for pigmented skin lesions in general practice,^15^ and the authors' randomised control trial evidence showed that GPs using it systematically can manage patients presenting with suspicious pigmented lesions safely and effectively.^16^ In mid-2016, an electronic version of the 7PCL was disseminated in a routine upload by a leading GP clinical system (EMIS) to more than 50% of UK GPs^17^ as an eCDS for melanoma (see Figure 1). In this study, the use of this eCDS for melanoma in routine primary care was explored qualitatively. The aim was to understand GP and patient perspectives on the implementation and usefulness of the melanoma eCDS compared with the paper version.

## Method

### Participants and study design

Two clinical research networks (CRNs) approached EMIS-using general practices. Four agreed to participate, and their GPs were reminded of where to locate the melanoma eCDS in their EMIS software. GPs were purposively sampled to ensure maximum variation in age, sex, years of experience, and teaching experience. Six to 8 weeks later, the researcher visited each practice to conduct interviews with these GPs.

During this visit the electronic health records were searched to identify all the practice patients who had presented with a skin lesion during the same time period and had the melanoma eCDS used during their consultation. Again, they were purposively sampled to ensure maximum variation in age, sex, and dermatology referral. If deemed eligible by their GP, patients were mailed study invitation packs, and invited to respond directly to the research team if they agreed to take part in an interview. Written informed consent was gained from the relevant GP or patient participant before the start of each interview.

### Data collection

In-depth semi-structured interviews were used to collect patient and GP views. Interviews were conducted face-to-face with GPs, and by telephone with patients. Development of the interview schedules was supported by literature searches and the expertise of the clinician team members. The patient representative member of the research team reviewed both the GP and patient interview schedules and made helpful improvements. The GP interview schedule explored introduction and engagement with the eCDS; general issues about melanoma diagnosis; and strategies around eCDS implementation. The patient interview schedule explored use of the eCDS tool within the consultation; patient recall of the use of the tool; perceptions about quality of care; and outcome of consultation, including any subsequent follow-up. All interviews were conducted by an experienced researcher, audio-recorded, professionally transcribed verbatim, and anonymised. GP interviews lasted 26 minutes (mean; range 16–36 minutes), while patient interviews lasted 16 minutes (mean; range 10–24 minutes).

### Data analysis

**Figure 2.**
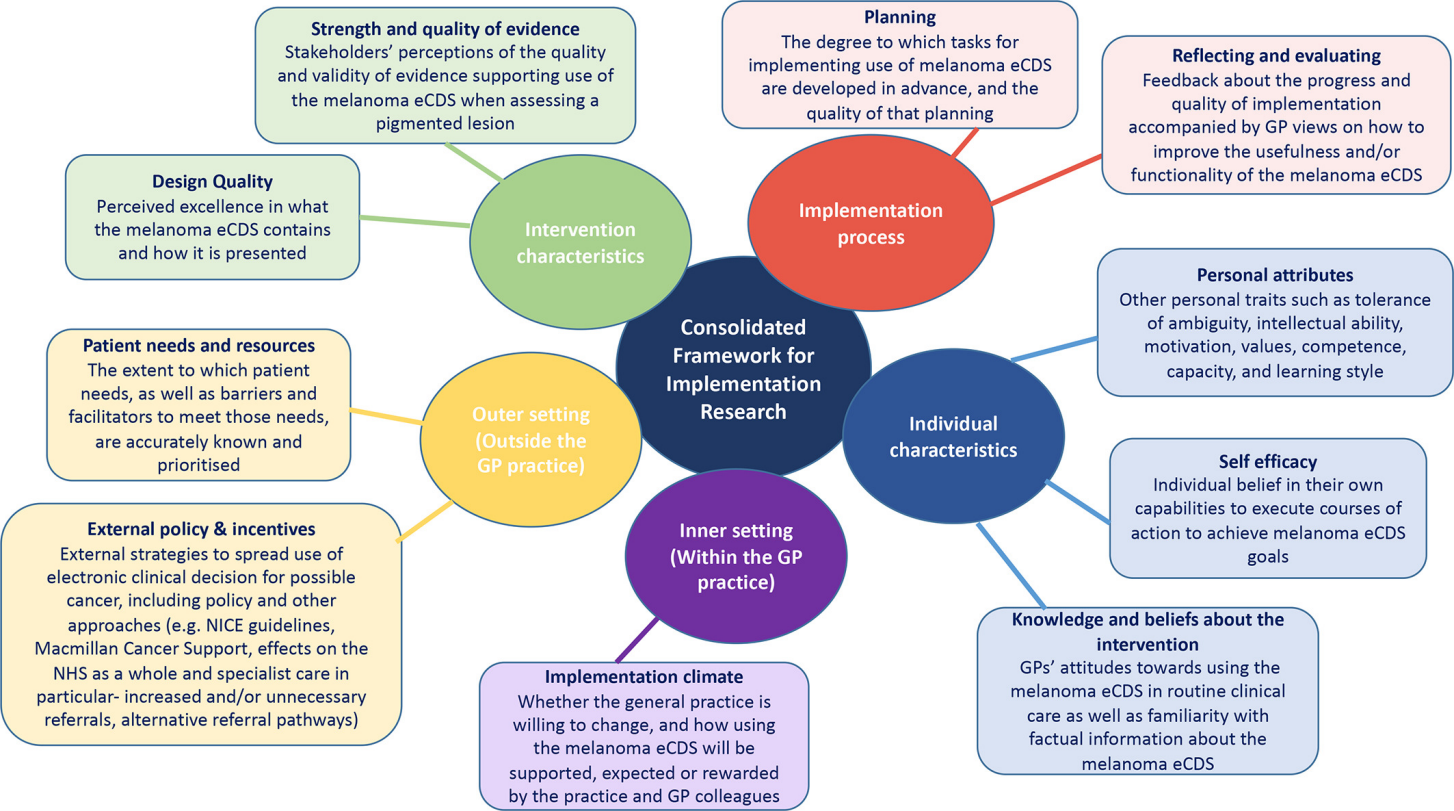
Overview of the Consolidated Framework for Implementation Research as applied to eCDS tools. eCDS = electronic clinical decision support.

The GP data were initially coded and categorised using a thematic approach to identify patterns or themes within the interview data.^18^ One researcher read each transcript several times, and coding began after familiarisation with the data. Descriptive themes were produced inductively based on the data. Most of the transcripts were also read by the clinical researchers, and this core research team met regularly to group the data into higher-level analytical themes, and to resolve any incongruences arising from the coding. As the thematic analysis progressed, the process was also informed by the CFIR, a model including five domains and further subthemes that affect the likelihood of an innovation being incorporated into routine clinical care.^19^ CFIR was developed from a meta-review of 12 systematic reviews, finding evidence for the domains and themes in the context of implementing clinical guidelines.^20^ The use of an iterative process was continued to sort the themes within the five main CFIR domains (see Figure 2); disconfirming data were also sought throughout.^21^

The patient data were summarised, but further themetatic analysis was deemed inappropriate due to the universal lack of awareness of the use of the melanoma eCDS during their consultations.

The data transcripts were organised and coded in NVivo (version 11). The presented quotations from the findings are the richest examples of the finalised analytical themes. GP quotations are accompanied by their sex and age, and patient quotations are accompanied by their sex, age, and whether the patient was referred to specialist care.

## Results

### Participant characteristics

Between August 2016–January 2017, 14 interviews were completed with GPs (9 female; mean age 44 years [range 27–60]; mean experience 17 years [range 1–33]), and 14 interviews with patients (11 female; mean age 63 years, [range 36–80]), six of whom were referred to a specialist. Characteristics of participating GPs and patients are presented in Table 1.

**Table 1A:** Characteristics of the participant GPs (*n *= 14)

	*n* (%)
**Age**	
Mean age, years (range)	44 (27–60)
≤40 years	5 (36)
41–50 years	4 (29)
≥51 years	5 (36)
**Sex**	
Female	9 (64)
Male	5 (36)
**Ethnicity**	
White British	11 (79)
Asian British	1 (7)
Mixed	1 (7)
Other ethnic group	1 (7)
**Years since qualification**	
Mean number of years (range)	17 (0–33)
≤5 years	5 (36)
6–25 years	5 (36)
≥26 years	4 (29)

**Table 1B:** Characteristics of the participant patients (*n *= 14)

	*n* (%)
**Age**	
Mean age, years (range)	63 (36–80)
<60 years	5 (36)
60–69 years	4 (29)
≥70 years	5 (36)
**Sex**	
Female	11 (79)
Male	3 (21)
**Ethnicity**	
White British	14 (100)
**Highest qualification**	
None	2 (14)
GCSE equivalent	7 (50)
A-level	1 (7)
Undergraduate degree	2 (14)
Postgraduate	1 (7)
PhD	1 (7)
**Employment status**	
Retired	9 (64)
Working	4 (29)
Homemaker	1 (7)
**Consultation outcome**	
Referral to a specialist	6 (43)
No further action	8 (57)

### GP perspectives

GPs' reflections could be mapped to all five domains of the CFIR, although most discussions focused on the intervention characteristics, outer setting, and individual characteristics (see Figure 2). Some quotations covered multiple themes within the CFIR framework, and therefore may be reported more than once.

#### 1. Intervention characteristics

##### 1.1 Strength and quality of evidence

The 2015 revised NICE guidelines for the management and referral of people with suspected cancer featured in all the GP interviews, and most mentioned the guidance to use the 7PCL to assess a mole. Several discussed how helpful it was when the 7PCL was integrated into 2-week wait urgent referral forms. However, some were concerned about their legal position once they considered a mole as a possible melanoma, and had used either the NICE guidelines or the melanoma eCDS:

*'If that’s the NICE guidance and that’s in the CCG 2-week wait form, if you’ve got a score of 4 and you don’t refer, I think the lawyers would say that you’re not following guidance and they could sue you.'* (F, 41–50 years)

##### 1.2 Design quality

Most were familiar with using the melanoma eCDS and felt that it was clear, useful, and easy to use. Some reflected on how using it did not intrude in a consultation, and that it could help with saving time during or after a consultation:

*'It’s simple to use and it’s on the referral form so it’s going to be easier to use it because then you can put the score on the form, speed things up really.'* (F, 41–50 years)

*'I think it's more time-efficient because it's tick, tick and you don't have to sit there and write "changed in size" and "changed in this" …'* (F, ≤40 years)

Most mentioned particular features of the melanoma eCDS that appealed to them or they found helpful, such as how it facilitated patient–GP communication in the consultation, and the ability to draw the shape and size of the lesion:

 '*… quite funky with the drawing ability on it'* (M, ≥51 years).

Some GPs reflected on how to improve the usefulness of the tool, including the addition of a pop-up prompt on the screen and a photograph for the clinical records:

*'I suppose the prompt of a photo to be added would be helpful if they need to look through it …'* (M, ≤40 years)

Some compared the melanoma eCDS favourably with other CDS systems:

*'It’s a lot simpler and quicker than QCancer. I’ve only used the QCancer once — I probably ought to have used it more, but it’s too long-winded to get your head around.'* (M, ≥51 years)

Several felt that expected features, such as family history or sun exposure, were missing, and a few felt that the melanoma eCDS was too simple and not useful:

*'I’m just concerned that it doesn’t ask about other things which are important, because maybe I would even refer a person who scores three, but has a lot of exposure* [or a] *strong family history.'* (F, 41–50 years)

#### 2. Outer setting (outside the GP practice)

##### 2.1 Patient needs and resources

Most GPs acknowledged patient concerns about possible melanoma and their wish for safe and accurate assessment in primary care, and rapid referral and diagnosis if the lesion appeared suspicious:

*'I think* [patients are] *very clued up on moles changes, skin cancer.'* (F, ≤40 years)

##### 2.2 External policy and incentives

Most GPs were positive about policy approaches to improve early diagnosis of all cancers in general, as well as melanoma in particular, but a few voiced doubts over the impact of early diagnosis on clinical outcomes:

*'Obviously it’s something to be encouraged, because if caught early then you’re going to prevent something that could become a lot more serious. The difficulty is we see an awful lot of pigmented lesions … I’m aware that the diagnosis of* [melanoma] *has gone up, whereas the overall mortality from it has not, and perhaps giving people a cancer diagnosis earlier,* [may have] *no impact on their overall outcome.'* (M, ≤40 years)

However, almost all the GPs expressed concerns that using the melanoma eCDS could lead to increased or unnecessary referrals, and were worried about the impact on specialist care. At the same time, many acknowledged that more referrals could lead to more diagnoses:

*'The danger is if we start referring too many people and they all turn out not to have melanoma, then actually we’ve clogged up the system and it’s more difficult for people who do have to be seen. But the counterargument is it may be that more people need to be seen to be picking up some extra cases.' (*M, ≤40 years)

Many GPs discussed the advantages of the urgent referral pathways, and some highlighted efficient local referral routes:

*'There's really good systems in place if you're concerned about something, the 2-week wait systems work efficiently, we've got a good referral form … we've got good support with the email photo system … and we get a reply back from the consultant within 24 hours, to say "yes it's fine* [or] *refer them".'* (F, 41–50 years)

#### 3. Inner setting (within the GP practice)

##### 3.1 Implementation climate

No GPs recollected any practice discussions about whether they should implement the melanoma eCDS, or how its use would be supported:

*‘It just appeared in EMIS' *(M, >51 years). 

Furthermore, there were no discussions around how GP colleagues could support its use. A few considered whether people other than GPs, such as practices nurses or non-clinical staff, could safely use the melanoma eCDS. Although they were felt to be technically capable, it was generally felt that a GP needed to make the clinical decision:

*'*[Practice nurses] *haven’t done the training and they haven’t done the years of looking at skin lesions that we get every day.'* (M, ≥51 years)

#### 4. Individual characteristics: knowledge, attitudes, self-efficacy, and other personal attributes

Most GPs were familiar with using the melanoma eCDS and felt that it was clear, useful, and easy to use. Some reflected on how it did not intrude in a consultation, and that it could help with saving time. There was a range of views about whether using the eCDS was of more value during the consultation to help with decision-making, or after the consultation for record-keeping, referral, and to confirm their management decision:

*'Without the checklist I already know what to look for. I know that if it’s changed in size, if it’s irregular, that those are all serious … So I would have already gone through it anyway, with or without the* [list] *in front of me, so does it really matter? Probably not. It’s in my head like any other medical problem, I mean, I consult all day long.' *(F, 41–50 years)

The GPs mainly felt that the melanoma eCDS was most useful for borderline decision-making:

*'My experience has been the scores have either been 5 or 6, or 1, and not much in the middle, so you either will or you won't* [refer] *… if there are lesions that score 2 or 3 or 4, that's going to be the grey area where actually it might help.'* (F, ≤40 years)

They also felt that it could be more useful for less experienced GPs or those who felt less confident when examining skin lesions:

*'I think that’s right for those … certainly trainees, and maybe younger GPs and people who haven’t done a lot of dermatology, yeah.'* (M, ≥51 years)

Some discussed using the melanoma eCDS for reassurance, either for themselves or for their patient:

*'… if someone was very worried and they scored zero then I might be able to say, “Look, this is a scoring system that’s been developed,” and it might just aid reassurance. Equally, if I was worried about someone and I wanted to explain why, I might just say, “Look, this is the scoring system, you’ve got quite a lot of points on this. It doesn’t mean it’s anything serious but it does mean we need to look into it more closely".'* (M, ≤40 years)

However, GPs were keen to discuss the importance of their clinical expertise and knowledge of their patients, and how they placed more value on their clinical judgment than the melanoma eCDS:

*'Our clinical knowledge still has to come through, you know, and it has to be tailored to individuals …'* (F, 41–50 years)

*'I’ve referred ones that didn’t score very highly. I’ve not referred ones that did score highly. I think they are helpful but I wouldn’t tie myself to them entirely.'* (M, ≤40 years)

Indeed, some showed clear antipathy to any form of guideline or checklist:

*‘ … checklists are for robots.’* (F, 41–50 years).

*'I think the downside of … making everything a tick-box exercise, does take away your clinical judgment, you can deskill. I don’t think that’s such a risk with this actually.'* (M, ≤40 years)

This appeared to be owing to the firmly held mental models for triaging patients’ symptoms and signs:

*'I think most GPs. … we are checklist people in our head, that’s what we’re trained to do and we would quite naturally tell the patient, “I think these are important, or these are not important, and I’m referring you because I am slightly worried that it is a bit irregular".'* (F, 41–50 years)

#### 5. Implementation process

##### 5.1 Planning

Most GPs discussed a lack of clear implementation strategies for guidelines and IT tools in their practice. Therefore, few felt that they had been made aware of it:

*'It may be that we* [partners] *just decide by email … Now it might probably go to the IT hub to decide on whether we should use it. But on the whole when these things arrive I think they tend to just get installed, especially something like templates which don’t force itself on you. You can still choose to make use of it. But the problem I had with that, we weren’t made aware of it.'* (M, ≤40 years)

##### 5.2 Reflecting and evaluating

There were no GPs or practices in this study that regularly monitored the impact on their referrals of using the eCDS or any other type of evaluation of impact. Some GPs reflected on how to improve the usefulness of the tool.

### Patient perspectives

The patients were universally unaware that the melanoma eCDS had been used during their consultation about a concerning pigmented lesion.

Table 2 contains illustrative quotations from each patient about their consultation, including whether they were examined, whether a checklist or computer tool was used, and whether they were referred. In summary, all the patients described their examination in detail, with half reporting that their GP had used a magnifying glass or dermatoscope to examine their skin. None recalled a checklist being used during the consultation, and most were very confident about this. Less than a third reported that the GP used a computer during their consultation, and all felt that this was for general data entry rather than for specific use of an electronic tool.

## Discussion

### Summary

In this qualitative investigation, the English GPs in the sample reported that, as the eCDS for melanoma was embedded in the electronic medical record, it was easy to use in routine clinical care, time-efficient, most useful for borderline clinical decision-making, could facilitate patient–GP communication in the consultation, and might improve timely diagnosis of melanoma and patient experiences. However, GPs were concerned that melanoma eCDS could lead to increased or unnecessary referrals to specialist care, and felt that better implementation could enhance its uptake and usefulness. Surprisingly, none of the 14 patients who were interviewed were aware that the melanoma eCDS had been used during their consultation with their GP, despite half being referred for a suspicious lesion and many GPs expressing the view that the melanoma eCDS could have most value when supporting borderline decision-making.

### Strengths and limitations

This is the first time that routine use in primary care of any electronic checklist or CDS tool for melanoma has been evaluated. The adoption of the widely used and rigorously developed CFIR model was chosen to support the analysis. This is the first time that it has been applied to an evaluation of an electronic checklist or CDS for possible cancer in the primary care setting, and it enabled the areas of improvement necessary to increase routine adoption of the eCDS for melanoma to be highlighted. Most of the GP interview data could be mapped onto the five domains of the CFIR model, although there were meagre data relating to the inner setting (implementation climate) or the implementation process. This was likely owing to the lack of explicit implementation by the computer software provider or the general practice managers. Nevertheless, uptake was probably better than expected owing to the melanoma eCDS simply being an electronic version of the NICE-recommended 7PCL, albeit with the addition of a drawing tool, enabling GPs to more accurately record the location of the lesion on their patient’s body. Using the CFIR model enabled these subtle distinctions to be drawn.

A further strength is that the GPs and patients were recruited from four general practices located across two CRNs in order to collect as wide a range of views as possible. Although there were more female than male GPs and most were white British, they represented a good range of ages and years in practice. It is possible that sex influenced attitudes towards the melanoma eCDS. Other limitations include the recruitment from only central and eastern England, and that patients were unaware of the eCDS use during their consultations. It is not clear whether the findings generalise to general practices, GPs and patients not involved in this research, or to those not using EMIS software. Also, the participating GPs may have held more extreme views than most GPs, being either more or less likely to have experienced implementation issues with the melanoma eCDS.

### Comparison with existing literature

CDS has been used in primary care for a range of acute and chronic conditions such as new-onset chest pain.^22^ It is also beginning to be used to support cancer screening^23^ and treatment decision-making. Few studies have examined the in-practice use of CDS to diagnose cancer earlier, although a randomised controlled trial set in English general practice is currently evaluating an eCDS tool for assessing upper gastrointestinal symptoms that could indicate cancer.^10^ One qualitative study explored English GPs’ experiences after embedding eCDS tools for suspected lung or colorectal cancer in primary care, and found them to be useful additions despite issues with 'prompt fatigue'.^14^ There have been more experimental primary care studies. In England, one examined the impact of a diagnostic decision support system on the consultation, and found that GPs felt that it changed their consultation style, by requiring them to code symptoms and signs while interacting with the patient, while patients sometimes commented that GPs were looking at their computer more than at them.^24^ Another study, using simulated consultations with Australian GPs to explore implementing a QCancer risk tool into general practice consultations, had similar findings; however, significant barriers were also found, most notably when the tool outcome and the experienced GP’s clinical judgment were not in line.^25^

### Implications for practice

A recent analysis of cancer registry data suggests, for the first time, that GP use of CDS may affect cancer outcomes as melanomas are thinner at diagnosis when referred by GPs with this eCDS embedded in their software compared with GPs without the eCDS embedded (Barclay M, *et al*, unpublished data, 2019). Therefore, there are two key implications from this study. First, while there is policy enthusiasm for integrating clinical decision rules into electronic health records, there are unanswered questions relating to the implementation and uptake of cancer eCDS in primary care settings. Successful implementation of a new tool, such as this melanoma eCDS, requires GPs to understand its value and how it can best be integrated into routine clinical practice. This study shows that this process needs to proceed with caution, and a great deal of attention needs to be tailored to implementation. These strategies are more likely to optimise implementation and equitable uptake by clinicians and patients alike. This is reflected in the literature; for example, while a systematic review of patient safety strategies targeted at reducing diagnostic errors by primary care clinicians found the strongest evidence for technology-based interventions, such as computer-assisted diagnostic aids and decision support algorithms,^26^ challenges to implementing the more widely used cardiovascular risk assessment tools were described.^27^ A recent review on risk prediction tools for cancer (for example, QCancer and risk assessment tools) found that, although there is a potential for clinical use, usability in the clinic by GPs and by patients at home is not well researched.^28^

Second, the 7PCL is already widely used by English GPs as a paper or ‘mental’ version. This study evaluated the use of an electronic version of the checklist rather than a more sophisticated algorithm-driven tool like QCancer, and found it to be useful, particularly for ‘borderline’ lesions. This is likely to have been owing to the immediate accessibility of the embedded eCDS in the medical records. While other technological approaches to help GPs assess skin changes or pigmented skin lesions, such as dermoscopy, are increasingly available, they currently lack evidence for their accuracy, safety, acceptability to clinicians and patients, and cost-effectiveness in the primary care setting. For these new technological approaches to reach their potential, proven (evidence-based) tailored implementation is of the highest importance.

In summary, most GPs in this study were familiar with the melanoma eCDS and felt that, compared with the paper version, it was easier to use, time-efficient, and could improve communication in the consultation. They were less clear that it could meet policy or patient needs to improve early diagnosis, and some felt that it could lead to unnecessary referrals. Few felt that it had been sufficiently implemented at practice level. More felt confident with their own management of moles, and that the eCDS could be most useful for borderline decision-making. For implementation to be successful, it should involve more than just installing new software on GPs’ computers overnight.

**Table 2.** Illustrative quotations from each patient about their consultation, including whether they were examined, whether a checklist or computer tool was used, and whether they were referred.

Patient ID, sex, age	Examination	Checklist use	Computer use	Referral Yes/No
01, F, <60 years	*'A junior doctor, she took some notes and she thought it was probably one of those keratosey things … she’d had a look through one of those little glass things that they put up, and she thought it was probably okay … Dr A came in to check what she’d said to me, he couldn’t look through one of those things and, because of my history, that’s why he sent me up'*	*'I don’t remember that, no'*	*'Yeah, she looked at it, asked me questions, I don’t remember them being questions on computer, it was more why was I worried about it…'*	Y
02, F, 60–69 years	*'She measured the dark brown marks and she looked at the others and said, “Yes, it had cleared up”'*	*'No'*	*'No, only to look up because she wasn’t the one who started the tablets off, to see how long I’d been taking them and no, not really'*	Y
03, M, 60–69 years	*'Yeah, she did … as I recall she looked at it, and then I think she just stretched the skin a little bit by it, you know, put her finger either side of it and just pulled it like that and looked at it again and … well I don’t think she asked me if I’d got any problems anywhere else, she just sort of felt my arms and then she said, “Well I think the best thing is to… get a specialist to look at it”'*	*'No, I didn’t* [notice]*'*	n/a	Y
04, F, ≥70 years	*'He took a photo and he looked at it, you know, with a sort of glass and he said "Well I think it's a small growth" and … I can't think what happened then… "We'll have to keep an eye on it", I think that's what he said'*	*'No, no.'*	*'Sometimes you can go there and see one and he never looks at you at all and he just types, but this one, he did sit and look at it, and then type it in, you know'*	Y
05, F, ≥70 years	*'She just recommended that I saw a dermatologist.'*	*'No'*	n/a	Y
06, F, ≥70 years	*'I obviously explained why I was there and that I’d been before and when, and he … tracked it down on a computer and found his notes from the previous consultation. And then he looked at it with a magnifying glass and he measured it and he … asked me if it was painful and if it was itchy and I’d already told him that I wasn’t sure but I thought it might have changed colour a bit and got a bit bigger'*	*'Right, well, I’m not aware of him using a specific tool, if … you’re really looking at the effectiveness of this tool'*	*'Yeah, he did use the computer during the consultation but they always do, don’t they? It didn’t seem that he used it any more or any less than normal'*	Y
07, F, <60 years	*'She had look at it and I think, I explained my history because I hadn’t seen this lady with my previous back thing, so she read the notes'*	*'No, I don’t remember that, no, we just talked about it and she read my notes'*	*'No'*	N
08, F <60 years	*'I asked her just to check them, so she had a look and she said they look absolutely fine, they do just naturally grow but she didn’t have any concerns with them at all … I think, yeah, just with her own eyes. I was going to say did she measure them but I don’t think she did. No, 'cos I really don’t think she was concerned about it.'*	*'No'*	*'No'*	N
09, M <60 years	*'Well, I just took my top off and she checked it, and then got, I know she looked through whatever it is they look through with their light or whatever, I don’t know what it’s called'*	*'Not really, no I don’t think so … to be honest I can’t remember, but I don’t think so.'*	*'No'*	N
10, M, <60 years	*'She looked at it by rubbing some funny gel on it and putting like a spy glass on it, I’m sure you’re familiar with that, I suspect it just aids the visibility. She informed me at that time that it was nothing problematic but offered to whip it off with some freezy spray next time I was in …'*	*'I don’t recall her looking at any, she didn’t give me any check sheets or computer stuff, no'*	*'I really can’t remember but I don’t think so'*	N
11, F, 60–69 years	*'He looked at it with a magnifying light and he did study it, yes, and I think he probably asked me how long I'd looked at it'*	*'No I don't think* [so]*'*	*'I don't think so'*	N
12, F, 60–69 years	*'I showed her and she had a look at it, she measured it with a little tool thing and she said she really couldn’t see anything wrong with it … I think she just measured it. It was a sort of a measuring device'*	*'No, no. I don’t think there was any need because it’s so unsinister'*	n/a	N
13, F, ≥70 years	*'I just explained the background, I said something about what was there and she had a look at it, and we spent enough time I felt with it, I was quite satisfied and I'm not sure what instrument she looked at it through, it was just a little black handheld thing that, you know, she looked at it very closely'*	*'No, no, because that wasn't the main thing I was really going there about so I guess she was running out of time too, but she did a good job, I was satisfied'*	n/a	N
14, F, ≥70 years	*'She had a look at them, she said there was only one that might possibly be and so the point of taking a photograph was to see if it got bigger because … that would be a sign that there might be something to worry about … We’ve done two further checks when I’ve been there for something else and both times she thought it hadn’t got bigger, this particular one that she’s keeping her eye on and photographing'*	*'No I’m quite sure she didn’t'*	n/a	N
